# How Notifications Affect Engagement With a Behavior Change App: Results From a Micro-Randomized Trial

**DOI:** 10.2196/38342

**Published:** 2023-06-09

**Authors:** Lauren Bell, Claire Garnett, Yihan Bao, Zhaoxi Cheng, Tianchen Qian, Olga Perski, Henry W W Potts, Elizabeth Williamson

**Affiliations:** 1 Department of Medical Statistics The London School of Hygiene and Tropical Medicine London United Kingdom; 2 Medical Research Council Biostatistics Unit University of Cambridge Cambridge United Kingdom; 3 Research Department of Behavioural Science and Health University College London London United Kingdom; 4 Department of Statistics and Data Science Yale University New Haven, CT United States; 5 Department of Biostatistics Harvard University Cambridge, MA United States; 6 Department of Statistics University of California Irvine Irvine, CA United States; 7 Institute of Health Informatics University College London London United Kingdom

**Keywords:** mobile health, mHealth, digital health, behavior change, behavior change, digital behavior change, engagement, micro-randomized trial, randomized trial, randomization, just-in-time adaptive intervention, adaptive intervention, push notification, notification, excessive alcohol consumption, smartphone app, alcohol, drinking, drinker, mobile phone

## Abstract

**Background:**

*Drink Less* is a behavior change app to help higher-risk drinkers in the United Kingdom reduce their alcohol consumption. The app includes a daily notification asking users to *“*Please complete your drinks and mood diary,*”* yet we did not understand the causal effect of the notification on engagement nor how to improve this component of *Drink Less*. We developed a new bank of 30 new messages to increase users’ reflective motivation to engage with *Drink Less*. This study aimed to determine how standard and new notifications affect engagement.

**Objective:**

Our objective was to estimate the causal effect of the notification on near-term engagement, to explore whether this effect changed over time, and to create an evidence base to further inform the optimization of the notification policy.

**Methods:**

We conducted a micro-randomized trial (MRT) with 2 additional parallel arms. Inclusion criteria were *Drink Less* users who consented to participate in the trial, self-reported a baseline Alcohol Use Disorders Identification Test score of ≥8, resided in the United Kingdom, were aged ≥18 years, and reported interest in drinking less alcohol. Our MRT randomized 350 new users to test whether receiving a notification, compared with receiving no notification, increased the probability of opening the app in the subsequent hour, over the first 30 days since downloading *Drink Less*. Each day at 8 PM, users were randomized with a 30% probability of receiving the standard message, a 30% probability of receiving a new message, or a 40% probability of receiving no message. We additionally explored time to disengagement, with the allocation of 60% of eligible users randomized to the MRT (n=350) and 40% of eligible users randomized in equal number to the 2 parallel arms, either receiving the no notification policy (n=98) or the standard notification policy (n=121). Ancillary analyses explored effect moderation by recent states of habituation and engagement.

**Results:**

Receiving a notification, compared with not receiving a notification, increased the probability of opening the app in the next hour by 3.5-fold (95% CI 2.91-4.25). Both types of messages were similarly effective. The effect of the notification did not change significantly over time. A user being in a state of *already engaged* lowered the new notification effect by 0.80 (95% CI 0.55-1.16), although not significantly. Across the 3 arms, time to disengagement was not significantly different.

**Conclusions:**

We found a strong near-term effect of engagement on the notification, but no overall difference in time to disengagement between users receiving the standard fixed notification, no notification at all, or the random sequence of notifications within the MRT. The strong near-term effect of the notification presents an opportunity to target notifications to increase “in-the-moment” engagement. Further optimization is required to improve the long-term engagement.

**International Registered Report Identifier (IRRID):**

RR2-10.2196/18690

## Introduction

### Background

Hazardous and harmful alcohol consumption is one of the major risk factors for many disease outcomes and poses a major public health burden [1,2]. Delivering brief interventions to reduce hazardous and harmful alcohol consumption is known to be effective [3]; however, such efforts are challenged by the sheer prevalence of harmful drinking and limited capacity of services [4,5]. There is a long-standing recognition of the need to broaden the reach of and access to brief, effective interventions to reduce harmful alcohol consumption for help-seeking individuals [6].

A promising solution is behavior change apps, as these are complex interventions that can capture dynamic patterns in human behavior and deliver support when an individual needs this the most [7-9]. Building on evidence that supports SMS text messaging as interventions to help individuals [10], behavior change apps can provide comprehensive, everyday support within people’s homes and diverse communities to maintain healthy behaviors [11]. However, a major concern is that insufficient engagement with an app is likely to hinder behavior change, particularly if a user disengages with the app soon after downloading it [12,13]. Engagement, a construct of both experiential and behavioral aspects [14], fluctuates within and between users over time and is influenced not only by the static content of the intervention but also by internal (eg, the user’s momentary mood, cognitive state, and recent patterns of engagement and drinking) and external (eg, the user’s current environment) factors [15-18].

Push notifications (reminders or pop-up messages on the screen) are often implemented to increase engagement with a behavior change app [13,19,20] and can have small, positive effects on engagement over a 24-hour period [21]. However, a more immediate causal effect (eg, within the next hour) of a push notification on engagement with behavior change apps has not yet been established [21,22]. We undertook a trial to estimate the causal effect of the notification on near-term engagement in the behavior change app *Drink Less* and to consider how the notification policy could be further optimized to improve engagement.

### The *Drink Less* App

*Drink Less* is a behavior change app that aims to help higher-risk drinkers in the UK adult population reduce their alcohol consumption. The app is freely available to people seeking help with their alcohol consumption, although it has not been advertised or targeted to specific groups of people. *Drink Less* was developed in line with the Medical Research Council guidelines for developing and evaluating a complex intervention [23-25] and the Multiphase Optimisation Strategy (MOST) framework [26,27] and is freely available on the Apple App Store. *Drink Less* is an evidence- and theory-informed intervention with several modules. The overall development and refinement of *Drink Less*, including how the behavior change modules were selected, can be found in previous publications [28,29]. The standard version of the app delivers a local daily notification at 11 AM, asking the user to “Please complete your mood and drinks diary” (Multimedia Appendix 1 provides a visual of the *Drink Less* notification). Daily notifications aim to remind users to self-monitor their drinking habits. The National Institute for Health and Care Excellence for the United Kingdom recommends self-monitoring as an effective technique for the act of noticing recent behavior and how this relates to their goals [30]. However, if a user has already engaged with the app to self-monitor their drinking that day, the notification may be an unnecessary reminder and may ultimately annoy the user over time.

The notification appears on the users’ notification center, and tapping the notification opens to the *Drink Less* landing page. The standard version of *Drink Less* sends a daily notification that aims to increase self-monitoring by tracking recent alcohol units consumed (ie, the day before). The delivery time at 11 AM allows users to complete their morning routines before engaging with the app. User feedback was received via the App Store, with the suggestion that a reminder to report drinking diaries in the evenings would be more helpful.

### Engagement With Drink Less

We previously reported exploratory research that visualized temporal patterns of engagement with *Drink Less* [31]. The visualizations showed limited depth of engagement, with 85% of sessions occurring within the Self-Monitoring and Feedback module, and a natural peak near 8 PM of both frequency (ie, number of log-ins) and time spent on the app observed in the evenings. This suggested that evenings are opportune moments to engage with *Drink Less* for longer sessions. In the evening, users may be more susceptible to harmful drinking and intervening at this moment of vulnerability and, at an opportune moment to engage, may be more conducive to reducing harmful patterns of drinking. In addition, our exploratory research discovered different trajectories of use, with 50% of users disengaging with the app 22 days after download. We hypothesized that a fixed notification policy may suit some users to maintain engagement, whereas other users may habituate to the daily notification policy and disengage sooner.

### Specific Aims and Objectives

We conducted a micro-randomized trial (MRT), a design in which users recruited for the MRT were repeatedly randomized to notifications over time, with outcomes measured after each randomization [32-36]. We aimed to provide evidence of how notifications affect near-term engagement as well as to consider further improvement of the push notification policy. The primary objective was to assess whether sending a notification at 8 PM increases behavioral engagement (opening the app) in the subsequent hour with *Drink Less*. The secondary objectives included the comparison of 2 different types of notifications. notifications. We also explored effect moderation by time and the exploration of effect moderation by user context (with context being a user’s dynamic state of engagement or habituation). We also aimed to understand the role of a notification policy more generally for time to disengagement. In addition, we aimed to compare 3 policies on time to disengagement (each policy being the decision rule of delivering notifications used in 1 of the 3 arms). This is the first step in our wider aspiration to optimize the notification policy of *Drink Less*, an aspiration we return to in the *Discussion* section.

## Methods

### Trial Design

Our study had a 30-day MRT with 2 additional parallel arms. Three different notification policies are implemented in the 2 arms and MRT to address the secondary objectives: (1) a standard policy of sending a daily message of *“Please complete your mood and drinks diary”* sent at 11 AM; (2) the MRT, a random policy that varies the content and sequence of the notifications; and (3) a no notification policy, a policy that does not send notifications. For the secondary objectives, the three policies are referred to as (1) standard notification policy, (2) random notification policy, and (3) no notification policy.

In total, 60% of eligible users were randomized to the MRT, and 40% of eligible users were randomized in equal number to the 2 parallel arms, either receiving the no notification policy or the standard notification policy of *“Please complete your mood and drinking diary”* at 11 AM.

For users randomized to the MRT, each user was randomized daily at 8 PM to receive 1 of the 3 options: no notification (with 40% probability), the standard message (with 30% probability), or a notification randomly selected with replacement from a bank of new messages (with 30% probability).

Following our MRT protocol [37] and the CONSORT (Consolidated Standards of Reporting Trials) 2010 guidelines [38], we reported the primary and some secondary results here.

### Participants

The recruitment period ran from January 2, 2020, to April 1, 2020. *Drink Less* is freely available on the Apple App Store, and individuals who downloaded the app during the recruitment period were eligible to participate in the trial if they self-reported a baseline Alcohol Use Disorders Identification Test (AUDIT) score of ≥8, which indicates excessive alcohol consumption [39]; resided in the United Kingdom; were aged ≥18 years; and reported being interested in drinking less alcohol.

The app prompted eligible users to read the privacy notice (Multimedia Appendix 2) and participant information sheet (Multimedia Appendix 3) before enrolling in the trial. During the informed consent process, users were informed that they could opt out of the trial at any time and that they would receive the standard version of the *Drink Less* app if they withdrew their consent.

The date of download is defined as the date when the onboarding process is completed by each user. The onboarding process involved users completing a registration section where they completed the AUDIT and sociodemographic assessment and then received normative feedback (personalized feedback on how their drinking compares with the behaviors of others). It is only after the completion of the onboarding process that users were then assessed for eligibility and consequently randomized to 1 of the 3 arms.

On enrollment in the study, we turned the permission function off within the app. This was intended to ensure that the participants received the notification policy to which they were randomized. Participants could, however, go into the settings and turn the notification policy off, which is applicable for all apps on the Apple App Store and is beyond the control of any app developer.

### Data

Preprocessing of the original use data was required. The raw engagement data are captured by a series of screen views, comprising time stamps of when a new screen is opened in the app. Clearing or *swiping away* the notification is not registered as any use [40]. The length of a session is calculated as the difference (in microseconds) between the first and last screen views, with a new session defined after 30 minutes of inactivity between screen views [41]. This method of calculating the length of sessions means that our measures of the length of time spent on the app are always underestimated because we do not know how long the user observes the last screen view [41]. We did not impose a threshold on our outcome (in terms of the amount or depth of app use), so simply opening the app is measured as engagement. When a user opens *Drink Less*, they are presented with a dashboard with various information about their drinking habits as well as a toolbox of features to access if they want. As such, simply opening the app and viewing the dashboard and toolbox present an opportunity for users to benefit from engaging with *Drink Less*. All time stamps were appropriately adjusted from Coordinated Universal Time to British Summer Time.

### Time-Fixed Measures (Baseline)

Time-fixed covariates, measured at baseline, were age, sex, type of employment (manual, nonmanual, or other), and baseline AUDIT score (0-40) [39,42,43]. The AUDIT risk zones were hazardous (8-15), harmful (16-19), and at risk for alcohol dependence (20-40). The participants self-selected the employment status they identified with for the options they were provided. They were not provided with a definition of employment type.

### Time-Varying Measures

Time-varying engagement measures within the MRT are the time stamps of when the user opens the app and the length of time (in seconds) spent on the app. This includes the time-varying variables: (1) “Did the user open the app before 8 PM on day of randomization? (yes/no)” and (2) “Did the user open the app any time after 9 PM the day before? (yes/no).”

Time-varying covariates, used as part of post hoc analyses to explore effect moderation, were “habituation” and “already engaged.” “Habituation” was captured using the binary measure “Did the user receive a notification the day before? (yes/no).” “Already engaged” was captured using the binary measure “Did the user open the app between 8 PM-9 PM the day before? (yes/no).”

### Interventions

The MRT tested 2 notification types. This trial tested the existing notification with the message of *“Please complete your mood and drinking diary”* and a new notification bank of 30 novel messages (Multimedia Appendix 4). The development of the new notification bank was informed by research with *Drink Less*, which found that the perceived usefulness of the app (the belief that using the app will help the user achieve their goal or goals and an indicator of users’ reflective motivation to engage) was associated with increased engagement for some users. Therefore, the new bank of notifications was designed (with feedback on the content sought from a group of behavioral scientists) to increase users’ reflective motivation to engage with a particular intervention module [44]. All messages contained the phrase “(using a particular module in the app) *can help you drink less*.” Examples include *“Recording if-then plans can help you drink less”* and *“Setting a doable goal can help you drink less. Take a moment to set a doable goal.”* The notification does not lock the user’s screen, and there is no expiry time for notification.

### Outcomes

The primary outcome was whether the user opened the app (yes or no) in the hour between 8 PM and 9 PM, following the randomization of receiving a notification at 8 PM. This is a time-varying, binary, and near-term measure of engagement.

We also defined a post hoc outcome of whether the user opened the app between 8 PM on the day of randomization to 8 PM the following day to explore the effect over a 24-hour period.

The secondary outcomes captured across the 3 different policies include the number of days to disengagement, with disengagement defined as the first day in a period of ≥7 consecutive days of no use.

### Sample Size

We powered the MRT for the important secondary objective of effect moderation over time, which guarantees at least as much power for the primary objective of detecting a marginal effect. Using a simulation informed by observational *Drink Less* data [31], we determined that a sample of 1200 users was sufficient to provide 80% power, with a type 1 error of 5%, to detect effect moderation over time, assuming a marginal notification effect of 2.16 decaying by 0.911 per day since download (Multimedia Appendix 5). We powered the secondary arms, implementing different notification policies, to detect a minimum absolute difference in time to disengagement of 10%, assuming 55% disengagement by day 22 under the standard policy compared with 65% under the no notification policy. To achieve 80% power with type 1 error of 5%, 372 users were required to receive each notification policy. This was rounded to 400 to simplify the randomization process. Overall, we aimed to recruit 1200 users to the MRT, 400 users to the standard notification policy, and 400 users to the no notification policy.

Previous download trends revealed, on average, an estimate of at least 33 eligible users per day who downloaded *Drink Less* and consented to the privacy notice. We expected the available recruitment window (January 2 to April 1, 2020) to be sufficient to reach our recruitment target of 2000 users. However, we fell short of this target of 2000 users and randomized 598 users in total for three reasons: (1) a large proportion of users (40%) did not give their informed consent to be part of the study; (2) the number of downloads, particularly for March 2020, was less than predicted, based on 2019 trends; and (3) extending the recruitment period to achieve the desired sample size was not possible because of the commencement of a prescheduled National Institute for Health Research–funded randomized controlled trial [45]. Consequently, the primary objective was sufficiently powered; however, we did not achieve the prespecified sample size for the secondary objectives of effect moderation over time and time to disengagement.

### Randomization

Simple randomization (unstratified and no blocking) was used. An external engineer generated the randomization sequence and coded it into the app. Together, 2 coauthors (LB and CG) pilot tested the randomization schedule. To further verify the randomization process, 10 volunteers also participated in a pilot test. The 10 volunteers were randomized into 3 different arms and asked to record the notifications received and the use of the app. We confirmed that the randomization process functioned as planned, and all uses were correctly captured.

### Statistical Methods Within the MRT

Descriptive statistics (frequency distributions and measures of central tendency) were used to describe the baseline variables of participants.

The primary outcome, within the MRT, was summarized separately for the standard notification, new notification, and no notification by the number of person-days where the app was opened between 8 PM and 9 PM then divided by the number of person-days in the MRT and expressed as a proportion.

The near-term effect of the notification on the primary outcome was expressed on the relative risk (RR) scale and pooled over the longitudinal data across all participants using the Estimator for the Marginal Excursion Effect (EMEE) [46]. The EMEE was developed to estimate the causal effects of time-varying treatments with binary outcomes. The EMEE does not require the correct specification of the marginal mean model (ie, how the time-varying engagement depends on a user’s time-varying contexts), providing robustness to highly complex and stochastic engagement patterns.

The effect of receiving a push notification versus not receiving a notification was estimated overall and then separately for the new message bank and standard notification. All models from the MRT were adjusted for the continuous variables of age, AUDIT score, days since download, the categorical variables of sex and employment type, and the time-varying variables “Did the user open the app before 8 PM that day?” and “Did the users open the app after 9 PM the day before?” The time-varying measures were included to increase the precision of our near-term notification effect, as they were likely highly correlated with the outcome. The covariates of “habituation” and “already engaged” are for the purpose of exploring how these recent states modify the near-term effect of the notification.

### Statistical Methods Across Arms

Baseline descriptive statistics and measures of use—the median number of sessions per user and the median length of sessions (seconds)—were reported across the 3 policies. A user was classified as having disengaged on the first day of a period of 7 consecutive days of no use. This outcome was only defined for the first 23 days after follow-up, which lasted 30 days in total. Survival curves were plotted using the Kaplan-Meier estimator [47] and compared using a log-rank test.

Owing to technical glitches, there were some unanticipated missing categorical baseline data. We reported the number of missing values for each arm. We used modal imputation for the baseline variables. To assess the sensitivity of our conclusions to our missing data approach, we imputed the data with the second most common value.

All analyses were conducted using R (version 4.0.5; R Foundation for Statistical Computing) [48] with the *dplyr* [49], *lubridate* [50], *gtsummary* [51], *zoo* [52], *ForImp* [53], and *survminer* [54] packages.

### Ethics Approval

Ethical approval for this study was granted by the University College of London’s Departmental Research Ethics Committee (CEHP/2016/556) on October 11, 2019, and The London School of Hygiene and Tropical Medicine Interventions Research Ethics Committee (17929) on November 27, 2019.

## Results

### Overview

**Figure 1:**
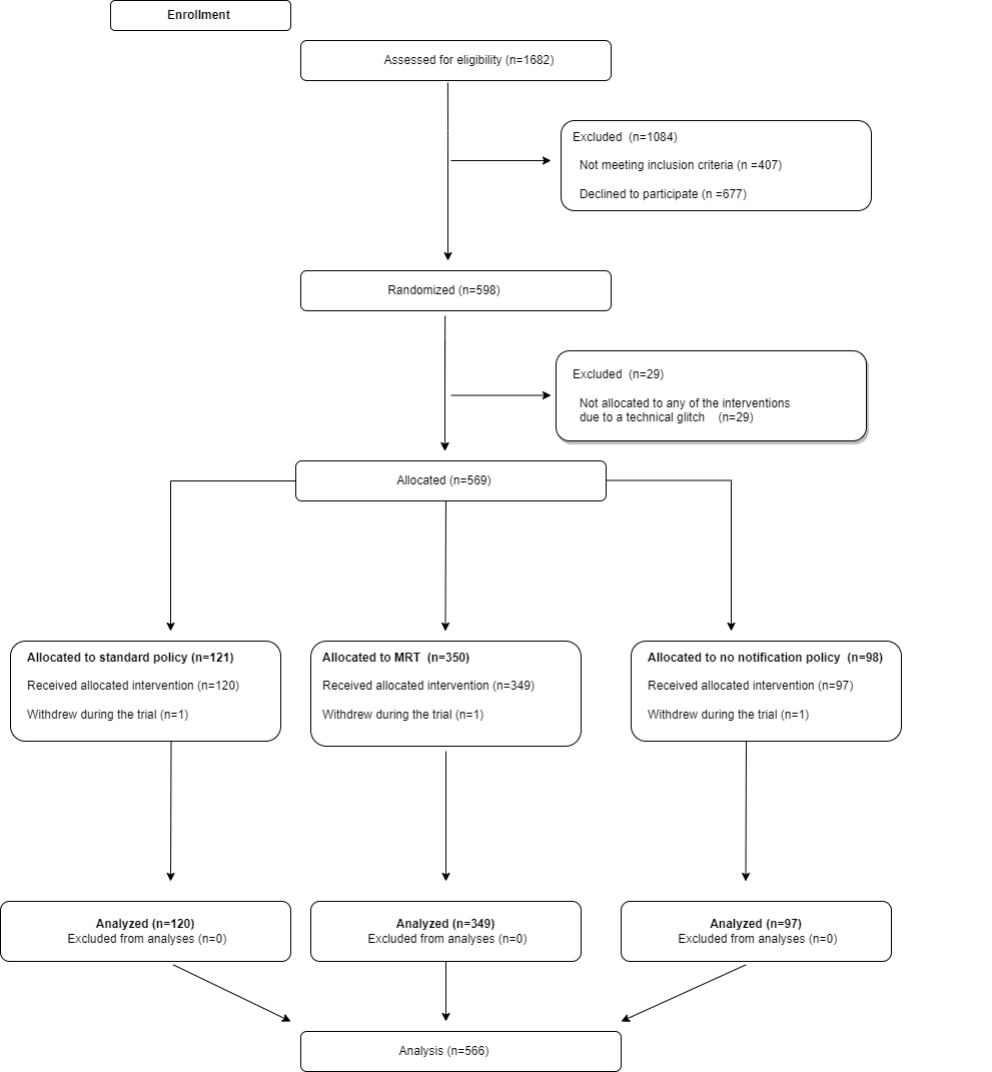
CONSORT (Consolidated Standards of Reporting Trials) flow diagram of the trial. MRT: micro-randomized trial.

The anonymized data sets, including data dictionaries, are publicly available [55]. The code for EMEE is openly available on the GitHub account [56]. Figure 1 presents the CONSORT flow diagram of the progress through the MRT.

### Recruitment

The trial recruitment period ran from January 2, 2020, to April 1, 2020 (app version 2.0.1). We analyzed a total of 566 users. The mean age was 44 (SD 12) years, with 43.6% (247/566) male and 45.8% (259/566) female users. A total of 62.4% (353/566) of users reported being in nonmanual employment, 12.5% (71/566) reported being in manual employment, and 14.5% (82/566) reported being in other employment types. A total of 48.8% (276/566) of users reported hazardous alcohol consumption, 20.1% (114/566) reported harmful alcohol consumption, and 31.1% (176/566) reported being at risk of alcohol dependence. A total of 68.2% (386/566) of users were disengaged by day 23 or earlier.

Data on sex and employment type were not recorded for the 60 users (standard arm, n=5; MRT, n=40; and no notification arm, n=5). We used modal imputation to set these missing values of sex to female and employment type as nonmanual (Table 1).

**Table 1:** Description of trial participants by randomized arm.

User characteristics		Baseline summary		
		Standard arm (n=120)	MRT^a^ (n=349)	No notification arm (n=97)
Age (years), median (IQR; n=566)		45 (35-55)	43 (34-51)	43 (34-52)
**Sex (n=506; standard arm: n=105; MRT: n=309; no notification arm: n=92), n (%)^b^**				
	Male, n (%)	43 (41)	155 (50.2)	49 (53)
	Female, n (%)	62 (59)	154 (49.8)	43 (47)
**Employment type (n=506; standard arm: n=105; MRT: n=309; no notification arm: n=92), n (%)^b^**				
	Nonmanual, n (%)	66 (62.8)	224 (72.5)	63 (68)
	Manual, n (%)	19 (18.1)	37 (12)	15 (16)
	Other, n (%)	20 (19)	48 (15.5)	14 (15)
**AUDIT score (n=566), n (%)**				
	Hazardous (8-15)	48 (40)	142 (40.7)	49 (51)
	Harmful (16-19)	29 (24.2)	84 (24.1)	18 (19)
	At risk of alcohol dependence (20-40)	43 (35.8)	123 (35.2)	30 (31)

^a^MRT: micro-randomized trial.

^b^Missing: standard arm: n=15; MRT: n=40; and no notification arm: n=5.

### Outcomes and Estimation

In the MRT, 349 users were randomized each day for 30 days, resulting in 10,470 measurements for the primary outcome. There were 30.05% (3146/10,470) of measurements for the new message, 29.72% (3112/10,470) of measurements for the standard notification, and 40.23% (4212/10,470) of measurements for no notification. The proportion of the primary outcome (opening the app between 8 PM and 9 PM) was 0.122 for the new message, 0.131 for the standard message, and 0.036 for no message. For the post hoc 24-hour outcome (from 8 PM to 8 PM the next day), the proportion of opening the app was 0.351 for the new message, 0.342 for the standard message, and 0.280 for no message.

### Main Results

Table 2 provides the results for the estimate of the near-term effect of the notifications on engagement. This demonstrates that, on average, the probability of opening *Drink Less* within the hour of receiving a notification increased 3.52-fold (95% CI 2.91-4.25). The 2 different notification types have similar effects, with the probability of opening *Drink Less* within the hour of receiving a standard notification increasing 3.66-fold (95% CI 2.99-4.48) and the probability of opening the *Drink Less* within the hour of receiving a new notification increasing 3.39-fold (95% CI 2.77-4.13).

Table 3 reports the results for how the effect of the notification changes over the first 30 days since download. We did not detect any significant changes over time, with an estimated decay by a factor of 0.993 per day with a 95% CI (0.975-1.012). Although not statistically significant, the point estimate of the decay in effect over time is larger in magnitude for the standard notification compared with the new notification; however, the wide CIs reflect large uncertainties in these estimates.

The summative engagement measures (median number of sessions and median length of sessions) under the 3 policies had a similar number of sessions and length of sessions over the first 30 days since download (Table 4). Users randomized to the no notification policy had, on average, slightly fewer but longer sessions, with a median of 43 seconds in length compared with 36 seconds for the standard policy and 35 seconds for the random policy (MRT).

**Table 2:** Primary objective—estimated near-term notification effect.

Notification type^a^	Relative risk (95% CI)
Pooled notifications (both standard and new)	3.523 (2.918-4.255)
Standard notification	3.664 (2.993-4.485)
New notification	3.385 (2.774-4.131)

^a^Adjusted for the continuous variables of age, Alcohol Use Disorders Identification Test score, days since download, the categorial variables of sex and employment type, and the time-varying variables “Did the use user the app before 8 PM that day?” and “Did the users use the app after 9 PM the day before?”

**Table 3:** Change of near-term notification effect over time.

Notification type^a^	Relative risk on the first day after download (95% CI)	Multiplicative change in effect (95% CI)
Pooled notifications (both standard and new)	3.849 (2.811-5.270)	0.993 (0.975-1.012)
Standard notification	4.193 (3.004-5.854)	0.989 (0.970-1.001)
New notification	3.534 (2.536-4.924)	0.997 (0.976-1.017)

^a^Multiplicative change in relative risk per day since download. Adjusted for the continuous variables of age, Alcohol Use Disorders Identification Test score, days since download, the categorical variables of sex and employment type, and the time-varying variables “Did the use user the app before 8 PM that day?” and “Did the users use the app after 9 PM the day before?”

**Table 4:** Median number of sessions per user and median length of sessions (seconds), across 3 arms for the first 30 days since download.

Policy implemented within arm	Number of sessions, median (IQR)	Length of sessions (seconds), median (IQR)
Standard policy	10.5 (4-23)	36 (9-115)
Random policy (MRT^a^)	9 (3-27)	35 (9-113)
No notification policy	6 (3-21)	43 (12-129)

^a^MRT: micro-randomized trial.

### Time to Disengagement—Survival Analysis

The median time to disengagement was 11 days for users randomized to the standard policy, 11 days for those randomized to the random policy (MRT), and 7 days for those randomized to the no notification policy. The number of disengaged users was 83 for the standard policy, 232 for the random policy (MRT), and 71 for the no notification policy. The log-rank *χ*^2^_2_ test statistic is 1.7, and the corresponding *P* value is .42.

**Figure 2:**
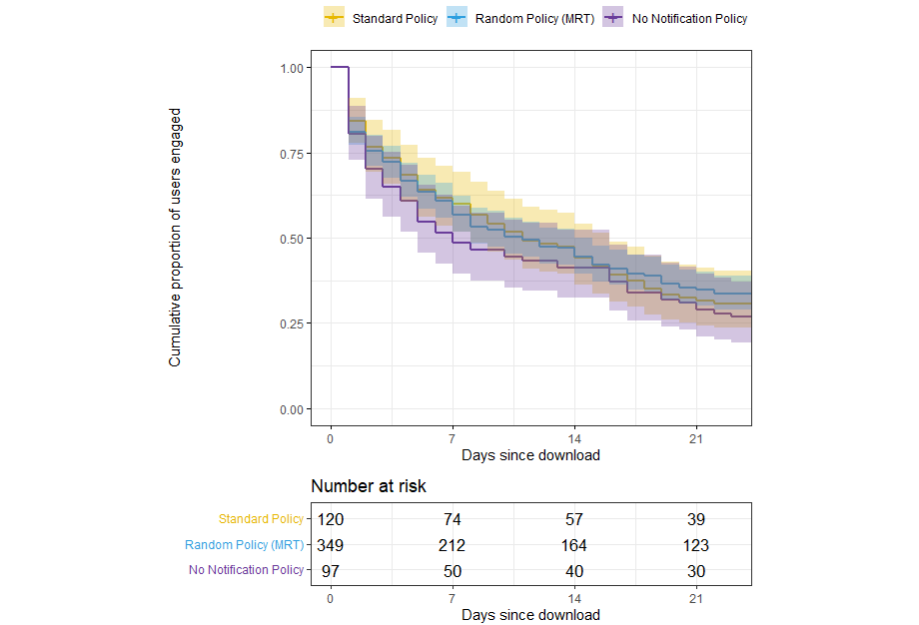
Kaplan-Meier plot of time to disengagement across the 3 notification policies. MRT: micro-randomized trial.

Figure 2 presents the Kaplan-Meier plot of time to disengagement across the 3 notification policies. This plot provides the estimated survival fraction over the first 30 days from the date of download between the 3 policies with 95% CIs. Although the survival fraction of the no notification policy may seem to accelerate at a faster rate than the other policies over the first week, all 3 policies had similar survival rates by day 23.

### Ancillary Analyses

Table 5 reports the estimates of the moderation of near-term notification effect, by recent engagement states, calculated from the MRT. We reported the estimated near-term notification effect for both message types pooled, and by notification type, and the effect is separated out by recent engagement states of habituation (yes or no) and recently engaged (yes or no). We reported the multiplicative difference in the notification effect, which demonstrates how the recent states of engagement modify the notification’s near-term effect. The near-term effect of both message types remains, and none of the estimates of effect moderation are statistically significant because of a limitation of the study lacking in power. If a user received a notification the day before, the near-term notification effect of a standard message is reduced by 11% (RR 0.889, 95% CI 0.60-1.31), whereas the effect of the new notification remains stable (RR 1.013, 95% CI 0.68-1.52). If a user is “already engaged” (they opened the app between 8 PM and 9 PM the day before), the near-term effect of the standard notification remained relatively stable (RR 0.97, 95% CI 0.65-1.42), whereas the near-term effect of the new notification decreased by 20% (RR 0.80, 95% CI 0.55-1.17).

The reported near-term notification effect for a 24-hour period is presented in Table 6. This demonstrates that notifications increase the probability of opening the app by 1.3-fold over a 24-hour period. Both notification types have a similar magnitude of effect.

**Table 5:** Estimated effect moderation by recent habituation and engagement.

Notification type^a^		Estimated notification effect		Estimated effect moderation
		Relative risk (95% CI)		Multiplicative difference in effect (95% CI)
		No	Yes	Ratio (yes or no)
**Habituation—“Did the user receive a notification the day before?”**				
	Pooled	3.645 (2.665-4.987)	3.449 (2.761-4.308)	0.946 (0.655-1.368)
	Standard	3.935 (2.837-5.458)	3.497 (2.739-4.465)	0.889 (0.601-1.314)
	New message	3.357 (2.381-4.732)	3.401 (2.692-4.298)	1.013 (0.677-1.517)
**Already engaged—“Did the user open the app between 8 PM and 9 PM the day before?”**				
	Pooled	3.620 (2.908-4.508)	3.168 (2.314-4.336)	0.875 (0.616-1.242)
	Standard	3.720 (2.938-4.710)	3.580 (2.542-5.043)	0.962 (0.652-1.420)
	New message	3.521 (2.794-4.439)	2.820 (2.015-3.946)	0.801 (0.548-1.168)

^a^All models are adjusted for the continuous variables of age; Alcohol Use Disorders Identification Test score; days since download; the categorical variables of sex and employment type; the time-varying variables “Did the users open the app before 8 PM that day?” and “Did the users open the app after 9 PM the day before?”; and the effect moderation variable of habituation.

**Table 6:** Post hoc analysis—estimated near-term notification effect, defined over 24 hours (from 8 PM to 8 PM the next day).

Notification type^a^	Relative risk (95% CI)
Pooled	1.260 (1.187-1.337)
Standard	1.245 (1.161-1.336)
New message	1.274 (1.193-1.360)

^a^Adjusted for the continuous variables of age, Alcohol Use Disorders Identification Test score, days since download, the categorial variables of sex and employment type, and the time-varying variables “Did the use user the app before 8 PM that day?” and “Did the users use the app after 9 PM the day before?”

## Discussion

### Principal Findings

We have shown that for *Drink Less*, there is a large near-term (3.5-fold) positive effect on engagement. The near-term notification effect for either the standard message type or a message from the new bank has similar effects on increasing engagement in the subsequent hour. Over a 24-hour period, a smaller, significant effect (1.3-fold) remained. We did not detect a significant change in the effects of notifications over time. The effect of receiving a new message that aims to reengage users was not significantly reduced by 20% if the user was already engaged. Furthermore, the effect of receiving a standard message was not significantly reduced by 12% if the user received a notification the previous day. There was no significant difference in (1) the mean number of days to disengagement, (2) the number of sessions, and (3) the length of sessions across the 3 different notification policies. However, a slightly longer median length of time was observed for a session under the no notification policy. One might hypothesize that unprompted behavioral engagement may include more attentive interest and cognitive investment.

In our study, despite evidence of a large positive notification effect on near-term engagement, an overall policy of sending a fixed daily notification or a random mix of notifications did not lengthen the time to disengagement or increase the amount of engagement during the first 30 days of since download. The results of the effect moderation analyses, although requiring confirmation in larger studies, suggest that notifications may be better served as dynamic interventions that adapt to a user’s fluctuating patterns of engagement. For example, by sending a notification to users when they are at an increased risk of disengagement, targeting them at that point with a notification intended to increase their perception of the usefulness of the app.

### Future Research to Optimize the Notification Policy

Our study demonstrated that for *Drink Less*, notification increases near-term engagement. This finding offers the opportunity for behavior change scientists to directly target the precise momentary states of an individual and to develop and implement dynamic theories for behavior change with *Drink Less.*

Efforts to maintain or increase engagement through consistent notifications could overburden or annoy a user, resulting in a state of disengagement with interventions from a previously motivated user [19]. Our findings suggest that the optimal role of notifications in improving long-term engagement is unlikely to be fixed or random components but better placed as dynamic components (ie, varying not randomly but in response to the user’s changing state of engagement and habituation)*.*

The open question now is when do we program notifications to be sent to balance goals of (1) intervening for maximum therapeutic effect based on a user’s internal history with *Drink Less* and external environmental factors; and (2) avoiding states of disengagement because of the burden of unhelpful notifications. To begin to answer this question, we will undertake further modeling of this MRT data to explore the within- and between-user effect of the notification over time and the balance of near-term and long-term effects. We will further analyze the data to understand if cue-to-action messages resulted in the task and to determine if the suggested module was engaged with. We imagine that a further optimized policy would (1) keep more users in a state of engagement for longer by sending fewer notifications than the random or fixed notification policies tested here, (2) have a higher near-term notification effect, and (3) ultimately improve the effectiveness of *Drink Less*. A type of machine learning called reinforcement learning may be helpful to personalize and optimizing the sequence of notifications over time [54,57,58]. The available data from our trial can provide a rich source of information to help guide the initial steps (ie, provide a “warm-start”) of the learning process of a reinforcement learning algorithm to improve engagement for *Drink Less* or other similar behavior change apps [57,59-61].

### Limitations

#### Overview

Our study was sufficiently powered for the primary objective, to detect a near-term notification effect. However, because we did not achieve our planned sample size, the important secondary objectives of effect moderation over time and time to disengagement between policies were not adequately powered. This resulted in wide CIs and large *P* values for the effect moderation analyses, leaving uncertainties about the existence and magnitude of these effects for the secondary objectives. Further studies with larger sample sizes are required to explore these effects.

There were missing data for a minority of the baseline values for sex and employment type, although our sensitivity analyses showed that the results were not sensitive to how the missing values were imputed.

The values entered for alcohol units consumed as diary entries were deemed too noisy to represent alcohol consumption over time for reasons of bias, extensive missing data, and backfilling (ie, users bulk reporting their drinking outcomes days later). Owing to the priority of not overburdening users with too many notifications sent within a day, our research does not provide a comparison of the near-term effect of the notification for different times of the day.

#### Generalizability

The recruitment period was from January 2 to April 1, 2020, which began with a typical surge in downloads in the new year and ended during the United Kingdom’s first COVID-19 lockdown. Such exogenous shocks to the users’ overall environment during the trial are likely to influence the underlying thoughts, emotions, and behaviors of reducing drinking levels (ie, people were mainly housebound) [62] and hence impact the patterns of engagement with *Drink Less*. The interpretation of the results is an average over this period only, with most of the recruitment occurring before the widespread outbreak in the United Kingdom (Multimedia Appendix 6). We also noted that the median time to disengagement in the standard policy arm (11 days) was much sooner than our data visualization cohort experienced (22 days; [31]).

### Conclusions

We found a large causal effect of sending notifications on near-term engagement. The probability of opening the app in the immediate hour increased 3.5-fold when receiving a notification compared with not receiving a notification. Notifications are important and effective components of behavior change apps; however, a policy of sending a fixed daily notification or a randomly chosen series of notifications did not increase the amount of engagement or length of time to disengagement for users compared with a policy of no notifications. This suggests that notifications may serve users better when they are implemented as dynamic components, such as sending a notification to increase the perceived usefulness of the app only when the users’ engagement patterns show that they are at risk of disengaging.

Further optimization of the notification policy is required to improve long-term engagement. The next stage of research is to explore how our findings would help develop a policy for *Drink Less* to intervene when a user is likely to benefit from support and keep more users engaged in the first 30 days after download.

## Appendix

Multimedia Appendix 1 Visual of *Drink Less* notification as it appears on the users’ notification center.

Multimedia Appendix 2 Privacy notice.

Multimedia Appendix 3 Information Sheet.

Multimedia Appendix 4 Bank of 30 newly developed messages and their link to the relevant behavior change module.

Multimedia Appendix 5 Sample size calculation.

Multimedia Appendix 6 Recruitment plots.

Multimedia Appendix 7 CONSORT-eHEALTH checklist (V 1.6.1).

## Abbreviations

AUDIT: Alcohol Use Disorders Identification Test
CONSORT: Consolidated Standards of Reporting Trials
EMEE: Estimator for the Marginal Excursion Effect
MOST: Multiphase Optimisation Strategy
MRT: micro-randomized trial
RR: relative risk
