# Supplementary figures and images for: How Notifications Affect Engagement With a Behavior Change App: Results From a Micro-Randomized Trial
Source: JMIR Mhealth Uhealth. 2023 Jun 9;11:e38342. doi: 10.2196/38342 (PMC10337295; doi:10.2196/38342)

Appendix 1: Visual of Drink Less modules and notification.


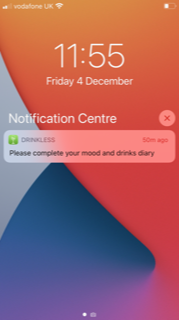

Supplement: Multimedia Appendix 1 [file mhealth_v11i1e38342_app1.docx]

#### Recruitment plots


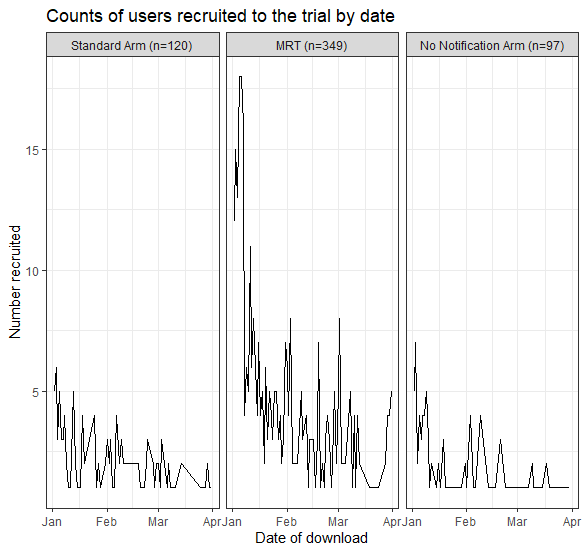

Supplement: Multimedia Appendix 6 [file mhealth_v11i1e38342_app6.docx]
