# Supplementary material for: How Notifications Affect Engagement With a Behavior Change App: Results From a Micro-Randomized Trial
Source: JMIR Mhealth Uhealth. 2023 Jun 9;11:e38342. doi: 10.2196/38342 (PMC10337295; doi:10.2196/38342)
Supplement: Multimedia Appendix 4 [file mhealth_v11i1e38342_app4.docx]

#### Bank of 30 newly developed messages and their link to the relevant behaviour change module.

| **Message content** | **Behaviour change module** |
| --- | --- |
| Tracking your drinks and days you don’t drink can help you drink less. | Goal Setting |
| Did you know that tracking your drinks and days you don’t drink can help you drink less? | Goal Setting |
| Tracking your drinks and days you don’t drink can help you drink less. Take a moment to track your drinks or a drink-free day. | Goal Setting |
| Setting a doable goal can help you drink less. | Action Planning |
| Did you know that setting a doable goal can help you drink less? | Action Planning |
| Setting a doable goal can help you drink less. Take a moment to set a doable goal. | Action Planning |
| Tracking your mood after drinking and drink-free days can help you drink less. | Self-Monitoring and Feedback |
| Did you know that tracking your mood after drinking and drink-free days can help you drink less? | Self-Monitoring and Feedback |
| Tracking your mood after drinking and drink-free days can help you drink less. Take a moment to track your mood. | Self-Monitoring and Feedback |
| Tracking your productivity levels after drinking and drink-free days can help you drink less. | Self-Monitoring and Feedback |
| Did you know that tracking your productivity levels after drinking and drink-free days can help you drink less? | Self-Monitoring and Feedback |
| Tracking your productivity levels after drinking and drink-free days can help you drink less. Take a moment to track your productivity levels. | Self-Monitoring and Feedback |
| Tracking your sleep quality after drinking and drink-free days can help you drink less. | Self-Monitoring and Feedback |
| Did you know that tracking your sleep quality after drinking and drink-free days can help you drink less? | Self-Monitoring and Feedback |
| Tracking your sleep quality after drinking and drink-free days can help you drink less. Take a moment to track your sleep quality. | Self-Monitoring and Feedback |
| Tracking how clear-headed you feel after drinking and drink-free days can help you drink less. | Self-Monitoring and Feedback |
| Did you know that tracking how clear-headed you feel after drinking and drink-free days can help you drink less? | Self-Monitoring and Feedback |
| Tracking how clear-headed you feel after drinking and drink-free days can help you drink less. Take a moment to track your clear-headedness. | Self-Monitoring and Feedback |
| Keeping an eye on how your drinking compares with others can help you drink less. | Normative Feedback |
| Did you know that keeping an eye on how your drinking compares with others can help you drink less? | Normative Feedback |
| Keeping an eye on how your drinking compares with others can help you drink less. Take a moment to check how your drinking compares with others. | Normative Feedback |
| Recording if-then plans can help you drink less. | Action Planning |
| Did you know that recording if-then plans can help you drink less? | Action Planning |
| Recording if-then plans can help you drink less. Take a moment to record an if-then plan. | Action Planning |
| Keeping an eye on which if-then plan has and hasn’t worked can help you drink less. | Action Planning |
| Did you know that keeping an eye on which if-then plan has and hasn’t worked can help you drink less? | Action Planning |
| Keeping an eye on which if-then plan has and hasn’t worked can help you drink less. Take a moment to check your if-then plans. | Action Planning |
| Playing the “yes please, no thanks” game can help you drink less. | Cognitive Bias Re-training |
| Did you know that playing the “yes please, no thanks” game can help you drink less? | Cognitive Bias Re-training |
| Playing the “yes please, no thanks” game can help you drink less. Take a moment to play the game. | Cognitive Bias Re-training |
