# Supplementary material for: How Notifications Affect Engagement With a Behavior Change App: Results From a Micro-Randomized Trial
Source: JMIR Mhealth Uhealth. 2023 Jun 9;11:e38342. doi: 10.2196/38342 (PMC10337295; doi:10.2196/38342)
Supplement: Multimedia Appendix 5 [file mhealth_v11i1e38342_app5.docx]

#### Sample size calculation.

Based on findings from the exploratory analyses of *Drink Less* users (manuscript submitted), we create generative models with a varying magnitude of treatment effect moderation with a few plausible values of marginal treatment effect. In particular, the exploratory analysis suggested that a crude estimate of the marginal treatment effect is 2.16 (in terms of relative risk), and the effect is modified by a factor of 0.911 for each additional day since download. Therefore, in the generative models, we let the marginal treatment effect to take values from 2.16, 1.80, and 1.50 (in terms of relative risk). For each marginal treatment effect value, we consider the magnitude of effect moderation by day-in-study (range –0.03 to –0.012) (in terms of log relative risk, assuming a log-linear model; eg, effect moderation–0.015 means that for each additional day in the study, the treatment effect decreases by a factor of exp(–0.015) = 0.985, and at the end of the study (day 30) the treatment effect will be exp(–0.015 × 29) = 0.647 of the treatment effect on the first day of the study). Figure 2 shows the result of the simulation-based sample size calculation, where the power to detect moderation for each sample size, marginal treatment effect, and moderation combination is calculated from 1000 simulations, where the critical value of the t-test is chosen to ensure 0.05 Type I error control.


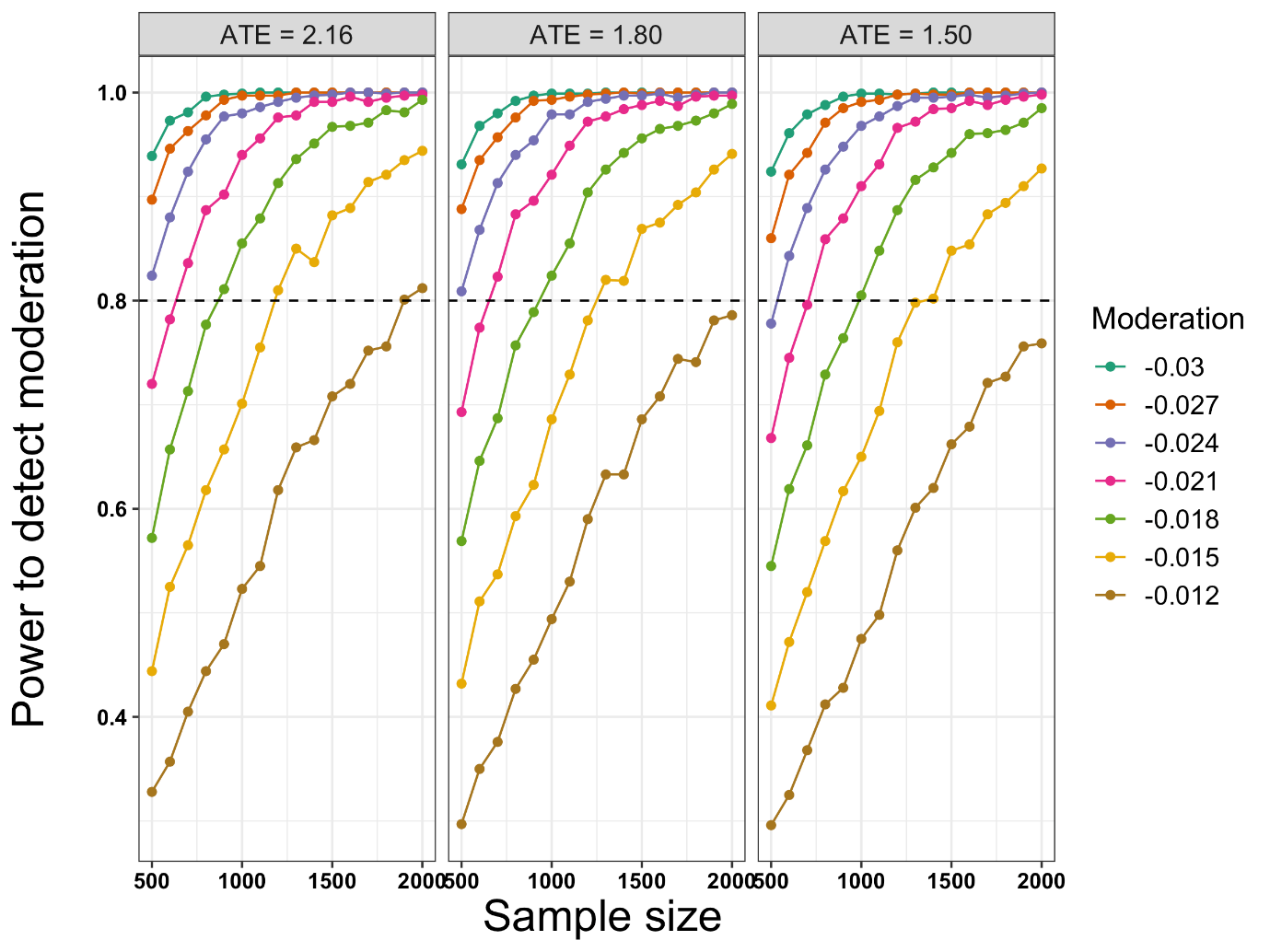


**Figure 2.** Result of the simulation-based sample size calculation. “ATE” (average treatment effect) denotes the causal excursion effect averaged over 30 days (relative risk scale). “Moderation” denotes the treatment effect moderation by day-in-study (on log relative risk scale, assuming a log-linear model; eg, effect moderation being –0.03 means that for each additional day in the study, the treatment effect decreases by a factor of exp(–0.03) = 0.97).

Simulations indicate that to ensure roughly 80% power to detect a –0.015 moderation (yellow curve in Figure 2) or larger moderation in magnitude, a sample size of 1200 is sufficient. Comparison across the three panels in Figure 2 shows that the power to detect moderation is relatively insensitive to different marginal treatment effect values. Because the sample size required to detect a nonzero marginal treatment effect is drastically smaller than the sample size required to detect moderation, we have close to 100% power to detect a nonzero marginal treatment effect under all of the sample sizes, marginal treatment effect, and moderation combinations considered in the simulation (results not shown). Therefore, we set the target sample size of the MRT arm to be 1200.

In the simulation-based sample size calculation, we consider the following generative model. The binary outcome (whether or not the user logged into the app or not between the hour of 8 PM to 9 PM) on day $t$ is generated from a Bernoulli distribution with success probability

$$\text{exp}\{\alpha_{0}+\alpha_{1}t+A_{t}\left( \beta_{0}+\beta_{1}t \right)\}$$

where $t$ denotes day-in-study (1, 2, …, 30), and $A_{t}$ is the binary indicator of whether a notification is sent on day $t$, with 0.6 probability to be 1 and 0.4 probability to be 0. Following (Qian et al., 2021) the marginal excursion effect averaged over time on relative risk scale (ie, “average treatment effect,” or ATE) is defined as

$$\text{ATE}=\frac{\sum_{t=1}^{30} \text{exp}(\alpha_{0}+\alpha_{1}t+\beta_{0}+\beta_{1}t)}{\sum_{t=1}^{30} \text{exp}(\alpha_{0}+\alpha_{1}t)}.$$

Using the result of the prior cohort data, in the generative model, we fix the success probability of the binary outcome under no treatment on day 1 to be 7.6%, and we fix the success probability of the binary outcome under no treatment on day 30 to be 2.2%. The effect moderation by day-in-study on log relative risk scale is $\beta_{1}$, which we vary between -0.030 and -0.012. We also vary ATE among three values: 2.16 (the crude estimate from the cohort data analysis), and two conservative values 1.80 and 1.50. Hence, for a given (ATE, β_1_) pair and the fixed success probability under no treatment on day 1 and day 30, we can compute the values of α_0_, α_1_, β_0_. The combination (α_0_, α_1_, β_0_, β_1_) determines the generative model.

Under each generative model, for a fixed sample size (500, 600, …, 2000), we simulate 1,000 data sets and calculate the *t*-statistic for testing $H_{0}:\beta_{1}=0$ using the EMEE estimator in Equation 10 of (Qian et al., 2021)by setting $S_{t}=(1,t)$, $g\left( H_{t} \right)=(1,t)$,  $\tilde{p}_{t}\left( S_{t} \right)=0.6$, and with a small modification in that the factor $\text{exp}\left( -A_{t}S_{t}^{T}\beta\right)$ is replaced by $\text{exp}\{-\left( A_{t}-0.6 \right)S_{t}^{T}\beta\}$. The *t*-statistic is formed by dividing the estimator of $\beta_{1}$ by its estimated standard error, and $H_{0}:\beta_{1}=0$ is rejected if the absolute value of the *t*-statistic is larger than 1.96. This ensures 0.05 Type I error control. Power under each setting is calculated as the proportion of simulations where $H_{0}$ is rejected.
