# Supplementary material for: How Notifications Affect Engagement With a Behavior Change App: Results From a Micro-Randomized Trial
Source: JMIR Mhealth Uhealth. 2023 Jun 9;11:e38342. doi: 10.2196/38342 (PMC10337295; doi:10.2196/38342)
Supplement: Multimedia Appendix 7 [file mhealth_v11i1e38342_app7.pdf]

CONSORT-EHEALTH Checklist V1.6.2 Report	Manuscript Number	38342
**(based on CONSORT-EHEALTH V1.6), available at [http://tinyurl.com/consort-ehealth-v1-6].**		
**Date completed**		
5/12/2023 7:50:07		
**by**		
Lauren Bell		
How notifications affect engagement. Results from a Micro-Randomized Trial		
TITLE		
**1a-i) Identify the mode of delivery in the title**		
"The Drink Less App<br>Drink Less is a behaviour change app that aims to help higher risk drinkers in the UK adult population reduce their alcohol consumption. The app is freely available to people seeking help with their alcohol consumption though the app has not been advertised or targeted to specific groups of people. Drink Less was developed in line with the Medical Research Council guidelines for developing and evaluating a complex intervention (Craig et al., 2008, Campbell et al., 2000, Skivington et al., 2021) and the MOST (Multiphase Optimisation Strategy) framework (Collins et al., 2007, Collins et al., 2014), and is freely available on the Apple App Store. Drink Less is an evidence- and theory- informed intervention with several modules. The overall development and refinement of Drink Less, including how the behaviour change modules were selected, can be found here (Garnett et al., 2019, Garnett et al., 2021b).		
**1a-ii) Non-web-based components or important co-interventions in title**		
" The standard version of the app delivers a local daily notification at 11 AM, asking the user to "Please complete your mood and drinks diary" (See Appendix 6 for a visual of the Drink Less notification). The daily notification aims to remind users to self-monitor their drinking. The National Institute for Health and Care Excellence (NICE) for the United Kingdom recommends self-monitoring as an effective technique for the act of noticing recent behaviour and how this relates to their related goals (Health and Excellence, 2014). However, if a user has already engaged with the app to self-monitor their drinking that day, the notification may be an unnecessary reminder and ultimately annoy the user over time. ""		
**1a-iii) Primary condition or target group in the title**		
" The recruitment period ran from 2nd January 2020 to 1st April 2020. Drink Less is freely available on the Apple App store, and individuals who downloaded the app during the recruitment period were eligible to participate in the trial if they self-reported a baseline Alcohol Use Disorders Identification Test (AUDIT) score of 8 or above which is indicative of excessive alcohol consumption (Allen et al., 1997); resided in the UK; were aged 18 years or over; and reported being interested in drinking less alcohol.<br>The app prompted eligible users to read the privacy notice (Appendix 2) and participant information sheet (Appendix 3) before proceeding to enroll in the trial. During the informed consent process, users were informed that they could opt out of the trial at any time and that they would receive the standard version of the app if at any time they withdrew their consent.<br>Upon enrolment to the study, we turned the permission function off within the app. This was with the intention to ensure that the participants received the notification policy they were randomised to. Participants could, however, go into the settings and turn the notification policy off, which is applicable for all apps on the Apple App Store and is beyond the control of any app developers.<br>"		
ABSTRACT		
**1b-i) Key features/functionalities/components of the intervention and comparator in the METHODS section of the ABSTRACT**		
"Background: Drink Less is a behaviour change app to help higher risk drinkers in the UK reduce their alcohol consumption. The app includes a daily notification, asking users to "Please complete your drinks and mood diary", yet we did not understand the causal effect of the notification on engagement nor how to improve this component of Drink Less. We developed a new bank of 30 new messages to increase users' reflective motivation to engage with Drink Less. In this study we aimed to determine how both the standard and new notifications affect engagement.<br>Objective: Our objective was to estimate the causal effect of the notification on near-term engagement, to explore whether this effect changed over time, and to create an evidence base to further inform optimisation of the notification policy.		
**1b-ii) Level of human involvement in the METHODS section of the ABSTRACT**		
Methods: We conducted a Micro-Randomised Trial (MRT) with two additional parallel arms. Inclusion criteria were Drink Less users who (1) consent to participate in the trial; (2) self-report a baseline Alcohol Use Disorders Identification Test score of 8 or above; (3) reside in the United Kingdom; (4) age ≥18 years and (5) report interest in drinking less alcohol. Our MRT randomised 350 new users to test if receiving a notification, compared to receiving no notification, increased the probability of opening the app in the subsequent hour, over the first 30 days since downloading Drink Less. Each day at 8 PM, users were randomised with 30% probability to receive the standard message, 30% probability to receive a new message or 40% probability to receive no message. To understand time-to-disengagement, 98 additional users were randomised to receive no notification and 121 users were randomised to receive the standard notification daily at 11 am. Ancillary analyses explored effect moderation by recent states of habituation and engagement.<br>Results: Receiving a notification, compared with not, increased the probability of opening the app in the next hour by 3.5-fold (95% confidence interval (CI) 2.91, 4.25). Both message types were similarly effective. The effect of the notification did not change significantly over time. A user being in a state of 'already engaged' lowered the new notification effect by 0.80 (95% CI 0.55, 1.16), though non-significantly. Across the three arms, time-to-disengagement was not significantly different.<br>Conclusion: We found a strong near-term effect of engagement on the notification but no overall difference in time to disengagement between users receiving the standard fixed notification, no notification at all, or the random sequence of notifications within the MRT. The strong near-term effect of the notification presents the opportunity to target notifications to increase 'in-the-moment' engagement. To improve longer-term engagement, further optimisation is required. "		
**1b-iii) Open vs. closed, web-based (self-assessment) vs. face-to-face assessments in the METHODS section of the ABSTRACT**		
". Our MRT randomised 350 new users to test if receiving a notification, compared to receiving no notification, increased the probability of opening the app in the subsequent hour, over the first 30 days since downloading Drink Less. "		
**1b-iv) RESULTS section in abstract must contain use data**		
To understand time-to-disengagement, 98 additional users were randomised to receive no notification and 121 users were randomised to receive the standard notification daily at 11 am. Ancillary analyses explored effect moderation by recent states of habituation and engagement.<br><br>Results: Receiving a notification, compared with not, increased the probability of opening the app in the next hour by 3.5-fold (95% confidence interval (CI) 2.91, 4.25). Both message types were similarly effective. The effect of the notification did not change significantly over time. A user being in a state of 'already engaged' lowered the new notification effect by 0.80 (95% CI 0.55, 1.16), though non-significantly. Across the three arms, time-to-disengagement was not significantly different. "		
**1b-v) CONCLUSIONS/DISCUSSION in abstract for negative trials**		
Conclusion: We found a strong near-term effect of engagement on the notification but no overall difference in time to disengagement between users receiving the standard fixed notification, no notification at all, or the random sequence of notifications within the MRT. The strong near-term effect of the notification presents the opportunity to target notifications to increase 'in-the-moment' engagement. To improve longer-term engagement, further optimisation is required.		
INTRODUCTION		
**2a-i) Problem and the type of system/solution**		
"Hazardous and harmful alcohol consumption is one of the major risk factors for many disease outcomes and has a significant global burden of health (Bagnardi et al., 2015, GBD 2016 Alcohol Collaborators, 2018). Delivering brief interventions to reduce hazardous and harmful alcohol drinking is known to be effective (Kaner et al., 2007) however such efforts are challenged by the sheer prevalence of harmful drinking and the limited capacity of services (Wilson et al., 2011, Kaner et al., 1999). There is a long-standing recognition of the need to broaden the reach of and access to brief, effective interventions to reduce harmful alcohol consumption for help-seeking individuals (Burton et al., 2017).<br>A promising solution is behaviour change apps, as these are complex interventions which can capture dynamic patterns in human behaviour and deliver support when an individual needs this the most (Fowler et al., 2016, Beckjord and Shiffman, 2014, Meredith et al., 2015). Building on evidence which supports short message services as interventions to help individuals (Bendtsen et al., 2021), behaviour change apps can provide comprehensive, every-day support, within people's homes and diverse communities, to maintain healthy behaviours (Satterfield, 2016). However, a major concern is that insufficient engagement with an app is likely to hinder behaviour change, particularly if a user stops engaging with the app not long after downloading it (Yardley et al., 2016, Alkhaldi et al., 2016). Engagement, a construct of both experiential and behavioural aspects (Perski et al., 2017), fluctuates within and between users over time, and is influenced not only by the static content of the intervention, but also by internal (e.g., the user's momentary mood and recent patterns of engagement and drinking) and external (e.g., the user's current environment) factors (Amoakoh et al., 2019, Chevance et al., 2021, Torous et al., 2020).<br>Push notifications (reminders or pop-up messages on the screen) are often implemented to increase engagement with a behaviour change app (Szinay et al., 2020, Alkhaldi et al., 2016, Milward et al., 2018) and can have small, positive effects on engagement over a 24-hour period (Bidargaddi et al., 2018). However, a more immediate causal effect (e.g., within the next hour) of a push notification on engagement with behaviour change apps is not yet established (Bidargaddi et al., 2018, Williamson et al., 2022).<br>"		
**2a-ii) Scientific background, rationale: What is known about the (type of) system**		

Push notifications (reminders or pop-up messages on the screen) are often implemented to increase engagement with a behaviour change app (Szinay et al., 2020, Alkhaldi et al., 2016, Milward et al., 2018) and can have small, positive effects on engagement over a 24-hour period (Bidargaddi et al., 2018). However, a more immediate causal effect (e.g., within the next hour) of a push notification on engagement with behaviour change apps is not yet established (Bidargaddi et al., 2018, Williamson et al., 2022).		
**Does your paper address CONSORT subitem 2b?**		
"Specific aims and objectives<br>The primary objective was to assess if sending a notification at 8 PM increases behavioural engagement (opening the app) in the subsequent hour with Drink Less. Secondary objectives included the comparison of two different types of notifications and the exploration of effect moderation by time or user's context. We also aimed to understand the role of a notification policy more generally for time-to-disengagement.<br>"		
METHODS		
**3a) CONSORT: Description of trial design (such as parallel, factorial) including allocation ratio**		
"Trial Design<br>Our study is a 30-day MRT with two additional parallel arms. Three different notification policies are implemented in the two arms and the MRT, to address secondary objectives. The different policies are (i) a standard policy of sending a daily message of "Please complete your mood and drinks diary" sent at 11 AM (ii) the MRT, a random policy which varies the content and sequence of the notifications, and (iii) a no-notification policy, a policy which no notifications are sent. For the secondary objectives, the three policies are referred to as (i) the standard notification policy, (ii) the random notification policy, and (iii) the no-notification policy.<br>Sixty percent of eligible users were randomised to the MRT, and forty percent of eligible users were randomised in equal number to the two parallel arms, either receiving the no notification policy or the standard notification policy, of "Please complete your mood and drinking diary" at 11 AM.<br>For users randomised to the MRT, each user was randomised daily at 8 PM, to receive one of the three options: no notification, the standard message, or a notification randomly selected with replacement from a bank of new messages. The randomisation probabilities for each day at 8 PM were 40% to receive no notification, 30% to receive the standard message and 30% to receive a randomly selected message (with replacement) from the bank of new messages.<br>Following our MRT protocol (Bell et al., 2020a) and the Consolidated Standards of Reporting Trials (CONSORT) 2010 guidelines (Schulz et al., 2010), we report the primary and some secondary results here. "		
**3b) CONSORT: Important changes to methods after trial commencement (such as eligibility criteria), with reasons**		
NA		
**3b-i) Bug fixes, Downtimes, Content Changes**		
Due to technical glitches, there was some unanticipated missing categorical baseline data. We report the number of missing values per arm. We used modal imputation for baseline variables. To assess sensitivity of our conclusions to our missing data approach, we imputed data with the second most common value.		
**4a) CONSORT: Eligibility criteria for participants**		
The recruitment period ran from 2nd January 2020 to 1st April 2020. Drink Less is freely available on the Apple App store, and individuals who downloaded the app during the recruitment period were eligible to participate in the trial if they self-reported a baseline Alcohol Use Disorders Identification Test (AUDIT) score of 8 or above which is indicative of excessive alcohol consumption (Allen et al., 1997); resided in the UK; were aged 18 years or over; and reported being interested in drinking less alcohol.<br>The app prompted eligible users to read the privacy notice (Appendix 2) and participant information sheet (Appendix 3) before proceeding to enroll in the trial. During the informed consent process, users were informed that they could opt out of the trial at any time and that they would receive the standard version of the app if at any time they withdrew their consent.<br>Upon enrolment to the study, we turned the permission function off within the app. This was with the intention to ensure that the participants received the notification policy they were randomised to. Participants could, however, go into the settings and turn the notification policy off, which is applicable for all apps on the Apple App Store and is beyond the control of any app developers.		
**4a-i) Computer / Internet literacy**		
NA		
**4a-ii) Open vs. closed, web-based vs. face-to-face assessments:**		
NA		
**4a-iii) Information giving during recruitment**		
All online - details in the appendix<br>The app prompted eligible users to read the privacy notice (Appendix 2) and participant information sheet (Appendix 3) before proceeding to enrol in the trial. During the informed consent process, users were informed that they could opt out of the trial at any time and that they would receive the standard version of the app if at any time they withdrew their consent.		
**4b) CONSORT: Settings and locations where the data were collected**		
The recruitment period ran from 2nd January 2020 to 1st April 2020. Drink Less is freely available on the Apple App store, and individuals who downloaded the app during the recruitment period were eligible to participate in the trial if they self-reported a baseline Alcohol Use Disorders Identification Test (AUDIT) score of 8 or above which is indicative of excessive alcohol consumption (Allen et al., 1997); resided in the UK; were aged 18 years or over; and reported being interested in drinking less alcohol.		
**4b-i) Report if outcomes were (self-)assessed through online questionnaires**		
The recruitment period ran from 2nd January 2020 to 1st April 2020. Drink Less is freely available on the Apple App store, and individuals who downloaded the app during the recruitment period were eligible to participate in the trial if they self-reported a baseline Alcohol Use Disorders Identification Test (AUDIT) score of 8 or above which is indicative of excessive alcohol consumption (Allen et al., 1997); resided in the UK; were aged 18 years or over; and reported being interested in drinking less alcohol.		
**4b-ii) Report how institutional affiliations are displayed**		
Not a required item		
**5) CONSORT: Describe the interventions for each group with sufficient details to allow replication, including how and when they were actually administered**		
**5-i) Mention names, credential, affiliations of the developers, sponsors, and owners**		
Stated in the acknowledgement section		
**5-ii) Describe the history/development process**		
The Drink Less App<br>Drink Less is a behaviour change app that aims to help higher risk drinkers in the UK adult population reduce their alcohol consumption. The app is freely available to people seeking help with their alcohol consumption though the app has not been advertised or targeted to specific groups of people. Drink Less was developed in line with the Medical Research Council guidelines for developing and evaluating a complex intervention (Craig et al., 2008, Campbell et al., 2000, Skivington et al., 2021) and the MOST (Multiphase Optimisation Strategy) framework (Collins et al., 2007, Collins et al., 2014), and is freely available on the Apple App Store. Drink Less is an evidence- and theory- informed intervention with several modules. The overall development and refinement of Drink Less, including how the behaviour change modules were selected, can be found here (Garnett et al., 2019, Garnett et al., 2021b). The standard version of the app delivers a local daily notification at 11 AM, asking the user to "Please complete your mood and drinks diary" (See Appendix 6 for a visual of the Drink Less notification). The daily notification aims to remind users to self-monitor their drinking. The National Institute for Health and Care Excellence (NICE) for the United Kingdom recommends self-monitoring as an effective technique for the act of noticing recent behaviour and how this relates to their related goals (Health and Excellence, 2014). However, if a user has already engaged with the app to self-monitor their drinking that day, the notification may be an unnecessary reminder and ultimately annoy the user over time.<br>The notification appears on the users' Notification Centre and tapping the notification opens to the Drink Less landing page. The standard version of Drink Less sends a daily notification that aims to increase self-monitoring through tracking of recent alcohol units consumed (i.e., the day before). The 11 AM time is to allow users time to complete their morning routines before engaging with the app. User feedback was received via the App Store, with a suggestion that a reminder to report drinking diaries in the evenings would be more helpful.		
**5-iii) Revisions and updating**		
NA		
**5-iv) Quality assurance methods**		
NA		
**5-v) Ensure replicability by publishing the source code, and/or providing screenshots/screen-capture video, and/or providing flowcharts of the algorithms used**		
NA		
**5-vi) Digital preservation**		
Opensource coding included in reference		
**5-vii) Access**		
Access via Apple App Store		
**5-viii) Mode of delivery, features/functionalities/components of the intervention and comparator, and the theoretical framework**		
NA		

**5-ix) Describe use parameters**		
NA		
**5-x) Clarify the level of human involvement**		
NA		
**5-xi) Report any prompts/reminders used**		
The study is about the optimization of the prompt		
**5-xii) Describe any co-interventions (incl. training/support)**		
NA		
**6a) CONSORT: Completely defined pre-specified primary and secondary outcome measures, including how and when they were assessed**		
Yes, consort guidelines and flow chart are mentioned		
**6a-i) Online questionnaires: describe if they were validated for online use and apply CHERRIES items to describe how the questionnaires were designed/deployed**		
NA		
**6a-ii) Describe whether and how “use” (including intensity of use/dosage) was defined/measured/monitored**		
NA		
**6a-iii) Describe whether, how, and when qualitative feedback from participants was obtained**		
NA		
**6b) CONSORT: Any changes to trial outcomes after the trial commenced, with reasons**		
The recruitment period ran from 2nd January 2020 to 1st April 2020. Drink Less is freely available on the Apple App store, and individuals who downloaded the app during the recruitment period were eligible to participate in the trial if they self-reported a baseline Alcohol Use Disorders Identification Test (AUDIT) score of 8 or above which is indicative of excessive alcohol consumption (Allen et al., 1997); resided in the UK; were aged 18 years or over; and reported being interested in drinking less alcohol.		
**7a) CONSORT: How sample size was determined**		
**7a-i) Describe whether and how expected attrition was taken into account when calculating the sample size**		
simulations study was performed and included in the paper		
**7b) CONSORT: When applicable, explanation of any interim analyses and stopping guidelines**		
Yes, consort guidelines and flow chart are mentioned		
**8a) CONSORT: Method used to generate the random allocation sequence**		
Simple randomisation in the app		
**8b) CONSORT: Type of randomisation; details of any restriction (such as blocking and block size)**		
simple (no blocking or stratification)		
**9) CONSORT: Mechanism used to implement the random allocation sequence (such as sequentially numbered containers), describing any steps taken to conceal the sequence until interventions were assigned**		
NA		
**10) CONSORT: Who generated the random allocation sequence, who enrolled participants, and who assigned participants to interventions**		
NA		
**11a) CONSORT: Blinding - If done, who was blinded after assignment to interventions (for example, participants, care providers, those assessing outcomes) and how**		
**11a-i) Specify who was blinded, and who wasn’t**		
No blinding		
**11a-ii) Discuss e.g., whether participants knew which intervention was the “intervention of interest” and which one was the “comparator”**		
Details in Appendix 1 and 2 -		
**11b) CONSORT: If relevant, description of the similarity of interventions**		
NA		
**12a) CONSORT: Statistical methods used to compare groups for primary and secondary outcomes**		
Yes - EMEE explained in stats section		
**12a-i) Imputation techniques to deal with attrition / missing values**		
Yes - sensitivity checks to primary outcome for missing baseline data		
**12b) CONSORT: Methods for additional analyses, such as subgroup analyses and adjusted analyses**		
Yes - adjusted variables stated.		
RESULTS		
**13a) CONSORT: For each group, the numbers of participants who were randomly assigned, received intended treatment, and were analysed for the primary outcome**		
Yes - flowchart provided		
**13b) CONSORT: For each group, losses and exclusions after randomisation, together with reasons**		
Yes - in flowchart		
**13b-i) Attrition diagram**		
NA		
**14a) CONSORT: Dates defining the periods of recruitment and follow-up**		
Yes, periods provided date recruited and follow measures and dates provided		
**14a-i) Indicate if critical “secular events” fell into the study period**		
Yes, lockdown dates addressed		
**14b) CONSORT: Why the trial ended or was stopped (early)**		
No- trial was not stopped early		
**15) CONSORT: A table showing baseline demographic and clinical characteristics for each group**		
Yes tables provided		
**15-i) Report demographics associated with digital divide issues**		
NA - only available for iPhone users		
**16a) CONSORT: For each group, number of participants (denominator) included in each analysis and whether the analysis was by original assigned groups**		
**16-i) Report multiple “denominators” and provide definitions**		
Yes - provided		
**16-ii) Primary analysis should be intent-to-treat**		
No relevant		
**17a) CONSORT: For each primary and secondary outcome, results for each group, and the estimated effect size and its precision (such as 95% confidence interval)**		
Yes provided		
**17a-i) Presentation of process outcomes such as metrics of use and intensity of use**		
Yes explored and raw data provided		
**17b) CONSORT: For binary outcomes, presentation of both absolute and relative effect sizes is recommended**		
Yes, provided		
**18) CONSORT: Results of any other analyses performed, including subgroup analyses and adjusted analyses, distinguishing pre-specified from exploratory**		
Yes, provided		
**18-i) Subgroup analysis of comparing only users**		
**19) CONSORT: All important harms or unintended effects in each group**		
Stated as not relevant		
**19-i) Include privacy breaches, technical problems**		
Stated some glitches		
**19-ii) Include qualitative feedback from participants or observations from staff/researchers**		
NA		
DISCUSSION		
**20) CONSORT: Trial limitations, addressing sources of potential bias, imprecision, multiplicity of analyses**		
**20-i) Typical limitations in ehealth trials**		

Limitations<br>Our study was sufficiently powered for the primary objective, to detect a near-term notification effect. However, due to not achieving our planned sample size, important secondary objectives of effect moderation over time and time to disengagement between policies were not adequately powered. This resulted in wide confidence intervals and large p-values for the effect moderation analyses, leaving remaining uncertainties about the existence and magnitude of these effects. Larger studies are required to explore these effects.<br>There was missing data for a minority of the baseline values of sex and employment type, though our sensitivity analyses showed that the result was not sensitive to how the missing values were imputed.<br>The values entered for alcohol units consumed as diary entries were deemed too noisy to represent alcohol consumption over time due to bias, extensive missing data and backfilling (i.e. users bulk reporting their drinking outcomes days later). Due to a priority to not overburden users with too many notifications sent within a day, our research does not provide a comparison of the near-term effect of the notification for different times of the day.		
**21) CONSORT: Generalisability (external validity, applicability) of the trial findings**		
**21-i) Generalizability to other populations**		
Principal Findings<br>We have shown that, for Drink Less, there is a large near-term (3.5-fold) positive effect on engagement. The near-term notification effect for either the standard message type or a message from the new bank have similar effects in increasing engagement in the subsequent hour. Over a 24-hour period, a smaller, significant effect (1.3-fold) remains. We did not detect a significant change in the effect of the notification over time. The effect of receiving a new message, which aims to re-engage users, was non-significantly reduced by 20% if the user was already engaged. Furthermore, the effect of receiving a standard message was non-significantly reduced by 12% if the user received a notification the day before. There was no significant difference in (i) the mean number of days to disengagement, (ii) number of sessions and (iii) length of sessions across the three different notification policies.		
**21-ii) Discuss if there were elements in the RCT that would be different in a routine application setting**		
**22) CONSORT: Interpretation consistent with results, balancing benefits and harms, and considering other relevant evidence**		
**22-i) Restate study questions and summarize the answers suggested by the data, starting with primary outcomes and process outcomes (use)**		
We found a large causal effect of sending a notification on near-term engagement. The probability of opening the app in the immediate hour increased 3.5-fold when receiving a notification, compared to not receiving a notification. Notifications are important and effective components of behaviour change apps; however, a policy of sending a fixed daily notification or a randomly chosen series of notifications did not increase the amount of engagement, or length of time to disengagement for users compared to a policy of no notifications. This suggests notifications may better serve users when they are implemented as dynamic components, such as sending a notification to increase the perceived usefulness of the app only when the users' pattern of engagement shows they are at risk of disengaging.		
**22-ii) Highlight unanswered new questions, suggest future research**		
Future research to optimise the notification policy<br>Our study has demonstrated that, for Drink Less, the notification increases near-term engagement. This finding offers the opportunity for behaviour change scientists to directly target the precise momentary states of an individual, to develop and implement dynamic theories for behaviour change with Drink Less. Efforts to consistently maintain or increase engagement could overburden or annoy a user, resulting in a state of disengagement with the interventions from a previously motivated user (Szinay et al., 2020). Our findings suggest that the optimal role of notifications to improve long-term engagement is unlikely to be fixed or random components, but better placed as dynamic components (i.e. varying not randomly but in response to the user's changing state of engagement and habituation).<br>The open question now is when do we programme notifications to be sent, to balance goals of (i) intervening for maximum therapeutic effect, based on a users' internal history with Drink Less and external, environmental factors; and (ii) avoiding states of disengagement due to the burden of unhelpful notifications. To begin to answer this question, we will undertake further modelling of this MRT data, to explore the within- and between- user effect of the notification over time, and the balance of near-term and long-term effects. We will further analyse the data to understand if cue-to-action messages resulted in the task, to determine if the suggested module was engaged with. We imagine a further optimised policy would (i) keep more users in a state of engagement for longer by sending fewer notifications than the policies tested here, (ii) have a higher near-term notification effect, and (iii) ultimately improve the effectiveness of Drink Less. A type of machine learning, called reinforcement learning, may be helpful to personalise and optimise the sequence of notifications over time (Zhu and Liao, 2017, Trella et al., 2022, O'Brien et al., 2022). The available data from our trial can provide a rich source of information to help guide the initial steps (i.e., provide a "warm-start") of the learning process of a reinforcement learning algorithm, to improve engagement, for Drink Less or other similar behaviour change apps (Zhu and Liao, 2017, Yao et al., 2021, Liao et al., 2020).		
Other information		
**23) CONSORT: Registration number and name of trial registry**		
International Registered Report Identifier (IRRID):<br>DERR1-10.2196/18690		
**24) CONSORT: Where the full trial protocol can be accessed, if available**		
Yes provided		
**25) CONSORT: Sources of funding and other support (such as supply of drugs), role of funders**		
Yes provided		
**X26-i) Comment on ethics committee approval**		
Yes, details of ethics approval provided		
**x26-ii) Outline informed consent procedures**		
Yes, in appendix provided is the informed consent and privacy notice		
**X26-iii) Safety and security procedures**		
Yes, in appendix provided is the informed consent and privacy notice		
**X27-i) State the relation of the study team towards the system being evaluated**		
NA		
